# miR‐224‐5p Suppresses Non‐Small Cell Lung Cancer via IL6ST‐Mediated Regulation of the JAK2/STAT3 Pathway

**DOI:** 10.1111/1759-7714.15516

**Published:** 2025-01-22

**Authors:** Jiao Tian, Yiming He, Zihui Zhang, Yuxin Zhu, Haixia Ren, Liang Zhang, Lei Li, Wei Li, Weidong Zhang, Ting Xiao, Honggang Zhou, Xiaoping Li

**Affiliations:** ^1^ Department of Thoracic Surgery, Tianjin First Central Hospital, School of Medicine Nankai University Tianjin China; ^2^ State Key Laboratory of Medicinal Biology, College of Pharmacy and Tianjin Key Laboratory of Molecular Drug Research Nankai University Tianjin China; ^3^ State Key Laboratory of Separation Membranes and Membrane Processes, Tianjin Key Laboratory of Green Chemical Technology and Process Engineering, School of Pharmaceutical Sciences Tiangong University Tianjin China

**Keywords:** IL6ST, JAK2/STAT3 signaling pathway, miR‐224‐5p, NSCLC

## Abstract

**Background:**

Our study aimed to explore the specific functions and potential mechanisms of miR‐224‐5p in non‐small cell lung cancer (NSCLC).

**Methods:**

We first analyzed the expression of miR‐224‐5p in NSCLC patients and cell lines through the GEO database and qRT‐PCR analysis. Then, we used MTT assays, wound healing assays, Transwell assays, and western blotting to evaluate the effects of miR‐224‐5p on NSCLC cell proliferation, migration, invasion, and epithelial–mesenchymal transition (EMT). Furthermore, we used a xenograft tumor model to evaluate the effect of miR‐224‐5p on NSCLC tumor growth. Potential binding targets of miR‐224‐5p were further identified through the target prediction databases, and the relationships between miR‐224‐5p, its targets, and downstream signaling pathways were further verified using luciferase reporter gene assays and western blotting.

**Results:**

The GEO database and qRT‐PCR analysis indicated that miR‐224‐5p was significantly downregulated in NSCLC patients and cell lines. Functional assays indicated that inhibiting miR‐224‐5p could enhance the proliferation, migration, invasion, and EMT of NSCLC cells, as well as accelerate tumor growth. In contrast, overexpression of miR‐224‐5p inhibited these processes. We identified IL6ST (interleukin 6 signal transducer) as a binding target of miR‐224‐5p. We observed that miR‐224‐5p could bind to and inhibit IL6ST expression and JAK2/STAT3 signaling pathway, and the inhibition of NSCLC tumor growth and JAK2/STAT3 pathway by miR‐224‐5p could be reversed by IL6ST overexpression.

**Conclusion:**

Our study demonstrated that miR‐224‐5p inhibited NSCLC by targeting IL6ST, thereby downregulating the JAK2/STAT3 signaling pathway.

## Graphical abstract

MiR‐224‐5p is significantly downregulated in non‐small cell lung cancer (NSCLC) patients and cell lines. MiR‐224‐5p inhibits NSCLC cell proliferation, migration, invasion, epithelial–mesenchymal transition (EMT), and tumor growth. MiR‐224‐5p targets IL6ST to suppress activation of the JAK2/STAT3 pathway, thereby inhibiting NSCLC progression.

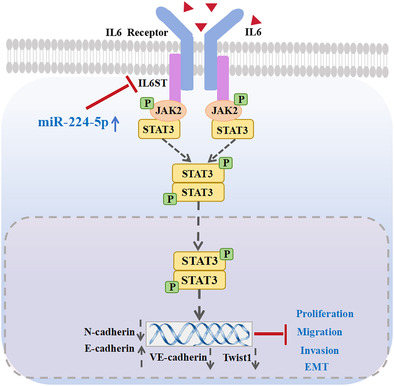

## 1 Introduction

Lung cancer is one of the most common and deadly malignancies worldwide, representing the leading cause of cancer‐related deaths in both men and women and accounting for 25% of all cancer deaths [1, 2]. Non‐small cell lung cancer (NSCLC) primarily comprises three subtypes: lung adenocarcinoma, lung squamous cell carcinoma, and large cell carcinoma [3], which together constitute approximately 85% of lung cancer cases [4]. The 5‐year survival rate of NSCLC varies depending on the stage at diagnosis, ranging from 63% for Stage I to only 6% for Stage IV [5]. The risk of recurrence remains high after traditional treatment, and the metastasis rate is also alarmingly high, with an average survival of less than 10–12 months [6]. Consequently, exploring and identifying effective targets for inhibiting NSCLC is crucial. Increasing evidence suggests that miRNAs play a crucial role in targeting and inhibiting NSCLC cells [7, 8].

MicroRNAs (miRNAs) are short, non‐coding RNAs that regulate gene expression after transcription [9, 10]. Their primary mechanism involves miRNAs binding to target mRNAs, leading to gene silencing. In stem cells, miRNAs play crucial roles in regulating proliferation, differentiation, and apoptosis. Imbalanced miRNA expression may disrupt cell signaling pathways and lead to diseases such as cancer [11, 12, 13]. Altered miRNAs can be predictive biomarkers for certain cancers [14]. Research has shown that restoring normal miRNA expression can significantly reduce tumor cell proliferation and invasion while inducing cancer cell senescence [15].

MiR‐224‐5p has been identified as a biomarker for several tumors [16], with some studies showing that miR‐224‐5p is expressed at low levels in a variety of tumors, such as melanoma [17], gastric cancer [18], glioma [19], acute myeloid leukemia [20], oral squamous cell carcinoma [21], and renal cell carcinoma [22]. However, the specific role of miR‐224‐5p in NSCLC is still unclear, and it is necessary to explore its function and mechanisms further. This study comprehensively examines the functions, targets, and molecular mechanisms of miR‐224‐5p in NSCLC.

## 2 Materials and Methods

### 2.1 Cell Lines and Cell Culture

A549 (adenocarcinoma cell), H226 (squamous cell carcinoma cell), H460 (squamous cell carcinoma cell), 95‐D (adenocarcinoma cell), and BEAS2B (normal lung epithelial cell) cells were sourced from ATCC and cultured in the recommended complete media. A549, NCI‐H226, NCI‐H460, and 95‐D cells were grown in RPMI‐1640 supplemented with 10% FBS (Gibco, USA) and 1% penicillin–streptomycin (Gibco, USA). BEAS2B cells were grown in DMEM with 10% FBS and 1% penicillin–streptomycin (Gibco, USA), and they are cultured under identical conditions at 37°C, 5% CO_2_.

### 2.2 Cell Transfection

Guangzhou Ruibo Biotechnology Co. Ltd. synthesized all miR‐224‐5p–related plasmids (miR‐224‐5p mimic and inhibitor). Before the experiment, A549, 95‐D, H460, and H226 cells were inoculated in 6‐well plates. The transfection assay was performed using Lipofectamine 2000 (Thermo Fisher Scientific, USA) according to the manufacturer's instructions for 48 h. Transfection efficiency was detected using RT‐qPCR.

### 2.3 MTT Assay

A549, H226, 95‐D, and H460 cell lines were seeded into 96‐well plates at 5 × 10^4^ cells per well. After 48 h of culture, MTT solution (20 μL/well) was added for 3.5–4 h at 37°C. Next, 150 μL of DMSO was added to dissolve the formazan, and the OD value (570 nm) was measure using a microplate reader (Thermo Scientific, USA). The experiment was repeated at least three times.

### 2.4 Wound Healing Assay

The treated cells were seeded in 24‐well plates, grown to 100% confluence, followed by treatment with mitomycin C (1 μg/mL) for 1 h. Then, a 100‐μm scratch was made with a sterile pipette tip, and the scratch was observed under an inverted optical microscope. The experiment was repeated three times. The cell migration distance was measured using ImageJ software.

### 2.5 Transwell Assay

The chamber used for the Transwell experiment was placed in a 24‐well plate. Cells were seeded into the chamber with pre‐cooled Matrigel and cultured for 24 h. After 24 h, the cells that migrated to the lower level of the chamber were fixed with 4% PFA and stained with crystal violet for 10 min. Finally, images were captured using an optical microscope (Nikon, Japan).

### 2.6 3D Culture Assay

Pre‐cooled Matrigel and serum‐free medium were mixed on ice at 1:3 ratio and added to a 96‐well plate, The plate was incubated at 37°C for 30 min. The cells were counted and inoculated into the plate with a total of 100 μL/well (5 × 10^4^ cells). After continued culture in the incubator for 24 h, the number of cells was recorded using an optical microscope (Nikon, Japan).

### 2.7 Western Blot Analysis

After 48 h of cell culture, the cells were lysed with RIPA buffer, and protein concentrations were measured by a BCA kit (Beyotime, cat# P0009, Shanghai). The normalized proteins were separated by SDS‐PAGE, incubating with primary antibodies against IL6ST (Affinity, cat#AF6291), phosphorylated JAK2 (p‐JAK2) (Tyr1007/1008) (CST, #3776), JAK2 (Affinity, cat# AF6022), phosphorylated STAT3 (p‐STAT3) (Tyr705) (CST, #4113), STAT3 (Affinity, cat# AF6294), and tubulin (Affinity, cat# AF7011) at 4°C overnight. The proteins were further incubated with secondary antibodies at room temperature for 2 h. Finally, chemiluminescent reagents were used to visualize the target proteins.

### 2.8 Luciferase Reporter Gene Assay

The IL6ST 3′‐untranslated region (3′‐UTR) luciferase reporter gene plasmid and the IL6ST mutant plasmid were co‐transfected into cells with miR‐224‐5p mimic and inhibitor using Lipofectamine 2000. Cell luciferase activity was then detected using the relevant luciferase detection kit (Promega, USA).

### 2.9 Immunofluorescence Assay

First, cells were seeded onto cell slides in 24‐well plates incubated for 48 h. Then, 4% paraformaldehyde (PFA) was used to fix the cells for 10 min and permeabilized with PBS containing 0.2% Triton X‐100 (Sigma‐Aldrich, USA) for 10 min. After blocking with 5% BSA for 60 min, the cells were incubated with primary antibodies overnight at 4°C. Fluorescein isothiocyanate (FITC)–labeled secondary antibodies were added and incubated for 2 h. Finally, the sections were mounted with a DAPI–containing mounting medium and examined under a confocal microscope (Leica, Germany) for imaging.

### 2.10 Nude Mouse Xenograft Model

Female BALB/c nude mice (5–6 weeks old, 18–22 g) were used in the animal experiment. All mice were fed an SPF barrier diet. H226 cells were stably transfected with the miR‐224‐5p mimic, A549 cells were transfected with the miR‐224‐5p inhibitor, and control cells (1 × 10^6^/mouse) were subcutaneously injected into the left anterior abdominal cavity of the nude mice to establish tumor models. The tumor volume of the nude mice was observed and recorded every other day, and the volume was calculated (*V* = *L* × *W* ^2^/2) for 21 consecutive days.

### 2.11 Immunohistochemistry

For immunohistochemistry (IHC) analysis, tumor tissue sections were first dewaxed, further hydrated with different concentrations of ethanol, and subjected to antigen retrieval with sodium citrate buffer. The sections were then blocked with PBS containing 0.2% Triton X‐100, 5% goat serum, and 3% H_2_O_2_ for 60 min at room temperature. Afterward, the sections were incubated with primary antibodies overnight at 4°C. IHC staining was performed the next day using DAB reagent conjugated with horseradish peroxidase (HRP), and nuclei were stained with hematoxylin. IHC images were captured using a microscope (Nikon, Japan).

### 2.12 Bioinformatics Analysis

GEO (Gene Expression Omnibus) datasets GSE63805 and GSE15008 were obtained from the NCBI database (https://www.ncbi.nlm.nih.gov/). Potential targets of miR‐224‐5p were predicted using the TargetScan (www.targetscan.org), miRanda (www.microrna.org), and PITA (http://genie.weizmann.ac) databases, with overlapping targets identified through the Venny online tool (http://tools/venny/index.html). The StarBase database (starbase.sysu.edu.cn) was used to assess binding sites and expression correlations between miR‐224‐5p and its targets. The GEPIA2 database (http://gepia2.cancer‐pku.cn) was utilized to examine IL6ST expression levels and generate survival curves in LUSC. Additionally, the cBioPortal database (https://www.cbioportal.org/) was used to evaluate the influence of target genes (ID3, PDE4DIP, IL6ST, and PIK3R3) on patient prognosis in LUSC.

### 2.13 Statistical Analysis

We used Prism Version 9.0 software for data analysis, and the data are expressed as mean ± SD. Differences were analyzed using a Student's *t*‐test. A one‐way analysis of variance was used to determine the significance of differences between multiple groups. A *p* value of < 0.05 was considered statistically significant (*p < 0.05, **p < 0.01, and ***p < 0.001).

## 3 Results

### 3.1 The Expression of miR‐224‐5p Is Decreased in NSCLC

**FIGURE 1:**
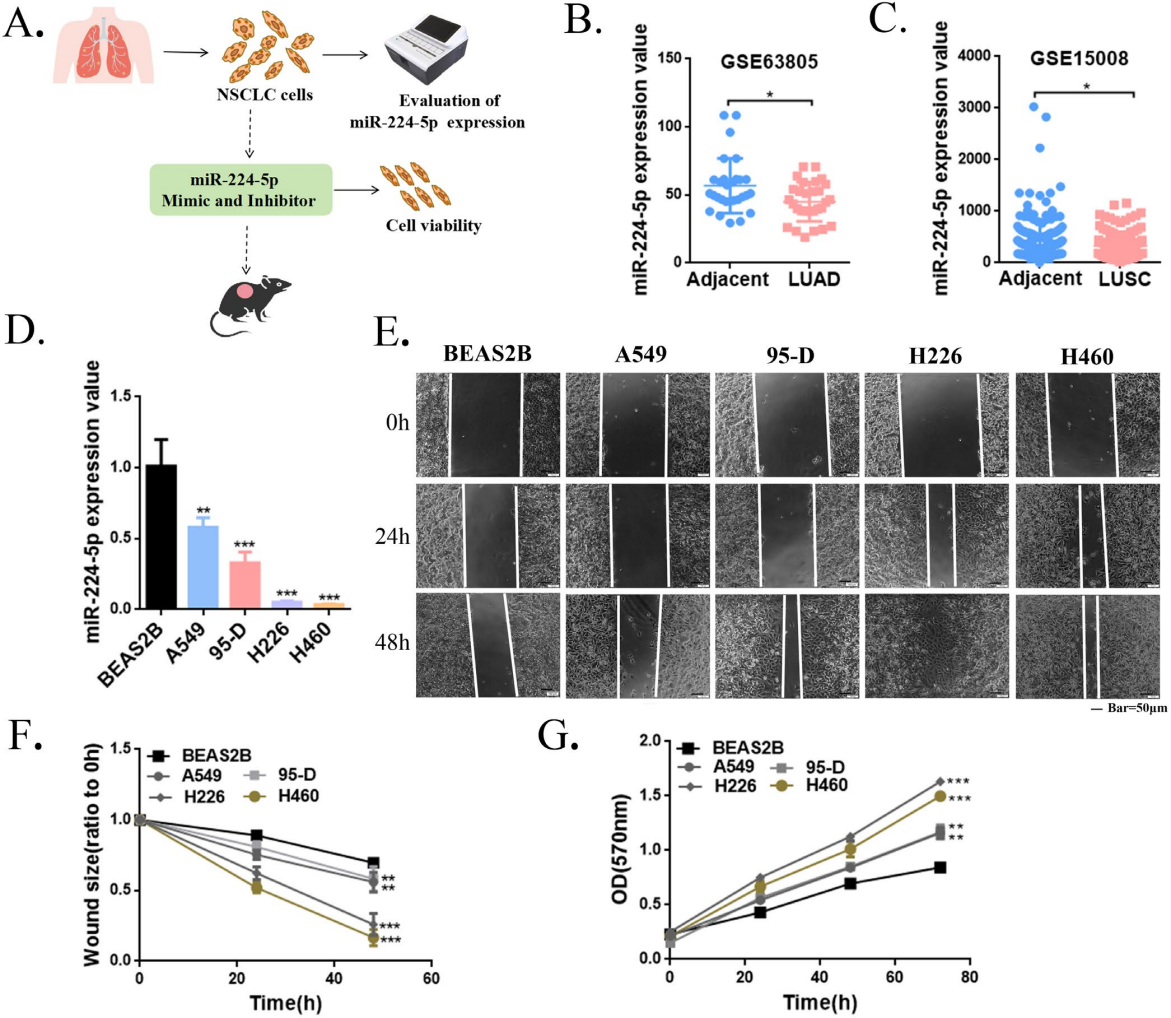
The expression of miR‐224‐5p is decreased in NSCLC. (A) A brief flowchart of this study. (B, C) Expression analysis of miR‐224‐5p in GEO datasets related to NSCLC patients (LUAD: lung adenocarcinoma; LUSC: lung squamous cell carcinoma). (D) Expression analysis of miR‐224‐5p in BEAS2B, A549, 95‐D, H226, and H460 cells by RT‐qPCR. (E, F) Wound healing assay was used to evaluate the differences in migration ability between different NSCLC cells and normal lung epithelial cells. (G) Evaluation of the differences in proliferation ability between different NSCLC cells and normal lung epithelial cells by MTT assay. The statistical significance was expressed as *p < 0.05; **p < 0.01; and ***p < 0.001. Values are expressed as mean ± standard error.

To explore the role of miR‐224‐5p in NSCLC progression, we first analyzed GEO datasets from GSE63805 and GSE15008. Analyzing the expression profiles of lung adenocarcinoma and squamous cell carcinoma patients in the dataset indicated that miR‐224‐5p expression was significantly decreased in cancer tissues compared with adjacent noncancerous tissues (Figure 1B,C). We then measured miR‐224‐5p expression in normal lung epithelial cells (BEAS2B) and four NSCLC cell lines (A549, H226, 95‐D, and H460) using qRT‐PCR. The results showed that miR‐224‐5p expression was reduced in all four NSCLC cell lines compared with BEAS2B. Notably, among these NSCLC cell lines, H226 and H460 had the lowest expression of miR‐224‐5p, while A549 and 95‐D had statistically significant and relatively high expression levels (Figure 1D). We performed a wound‐healing assay to evaluate the effect of miR‐224‐5p on migration of these cell lines. The results indicated that the migration ability of lung cancer cells was significantly higher than that of normal lung epithelial cells. In addition, cells with lower miR‐224‐5p expression levels (H226 and H460) migrated faster, while cells with higher miR‐224‐5p expression levels (A549 and 95‐D) migrated more slowly (Figure 1E,F). Furthermore, to evaluate cell proliferation, we performed a cell proliferation curve analysis, and the results showed that cells with lower miR‐224‐5p expression levels proliferated faster than cells with higher miR‐224‐5p expression levels (Figure 1G). The above results preliminarily suggest that the expression level of miR‐224‐5p is reduced in NSCLC, and miR‐224‐5p may play an essential role in the progression of NSCLC.

### 3.2 miR‐224‐5p Regulates NSCLC Cell Proliferation, Migration, Invasion, VM Formation, and EMT

**FIGURE 2:**
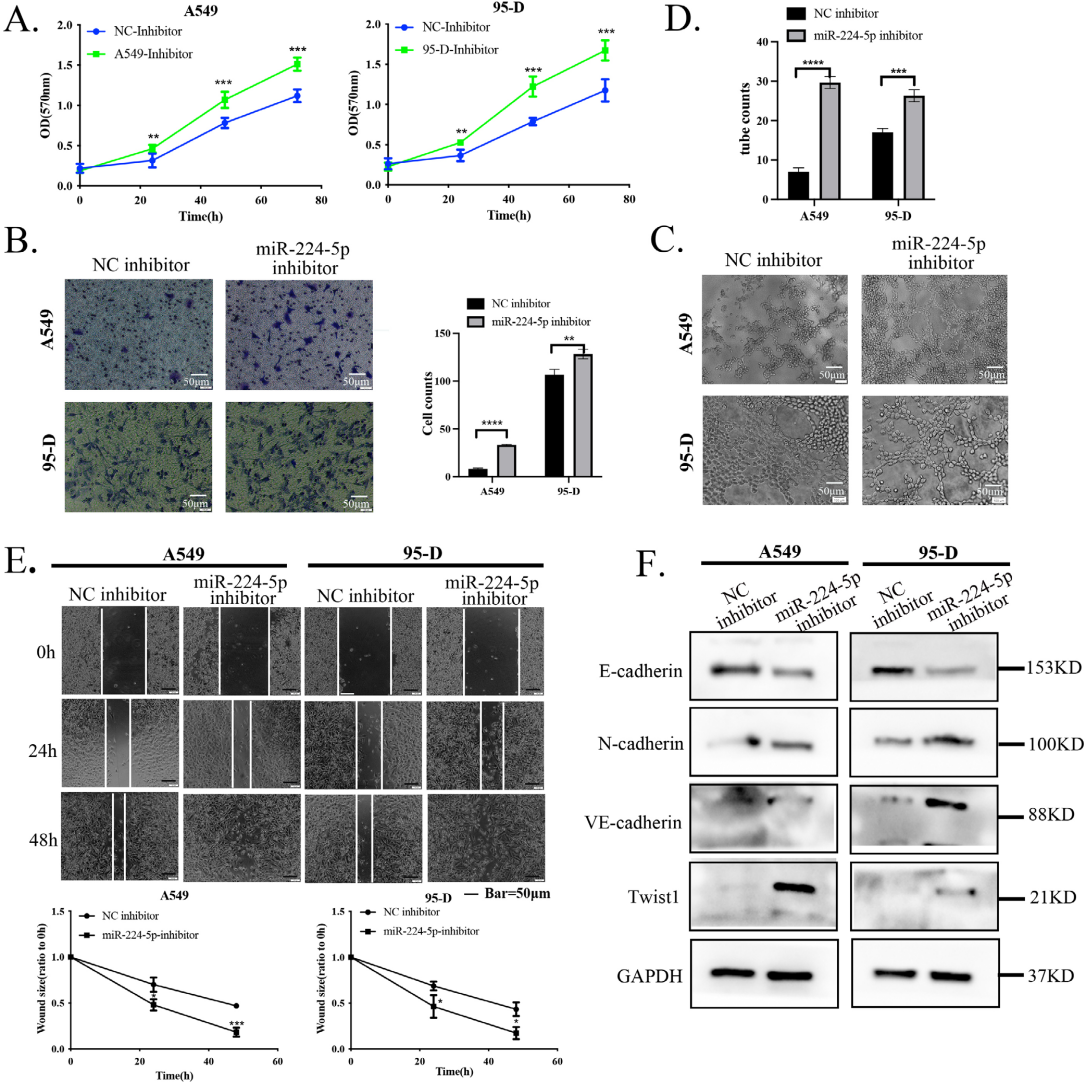
Inhibition of miR‐224‐5p promotes NSCLC progression. (A) Evaluation of the differences in the proliferation ability of A549 and 95‐D cells after introducing the miR‐224‐5p inhibitor using MTT assay. (B) The Transwell method evaluates the differences in the invasion ability of the above four types of cells. (C, D) Evaluation of the VM formation ability of A549, 95‐D cells, and cells after introducing the miR‐224‐5p inhibitor using 3D culture assay. (E) Evaluation of the differences in the migration of the four types of cells after treatment with mitomycin C using a wound healing assay. (F) Analysis of the expression levels of E‐cadherin, N‐cadherin, VE‐cadherin, and Twist1 proteins in A549 and 95‐D cells after introducing the miR‐224‐5p inhibitor by western blotting. The statistical significance was expressed as *p < 0.05; **p < 0.01; and ***p < 0.001. Values are expressed as mean ± standard error.

To further clarify the specific role of miR‐224‐5p in NSCLC, we transfected A549 and 95‐D cells with a miR‐224‐5p inhibitor to suppress miR‐224‐5p expression, and overexpressed miR‐224‐5p in H226 and H460 cells. After inhibiting miR‐224‐5p, we observed that A549 and 95‐D cells showed increased proliferation ability compared to control cells (Figure 2A). In addition, Transwell assay results indicated that A549 and 95‐D cells showed increased invasion ability after inhibiting miR‐224‐5p (Figure 2B). Three‐dimensional culture experiments also revealed that the tube‐forming ability of cells was significantly enhanced after inhibiting miR‐224‐5p (Figure 2C,D). Furthermore, we observed through cell wound healing assay that inhibiting miR‐224‐5p promoted the migration ability of A549 and 95‐D cells (Figure 2E).

**FIGURE 3:**
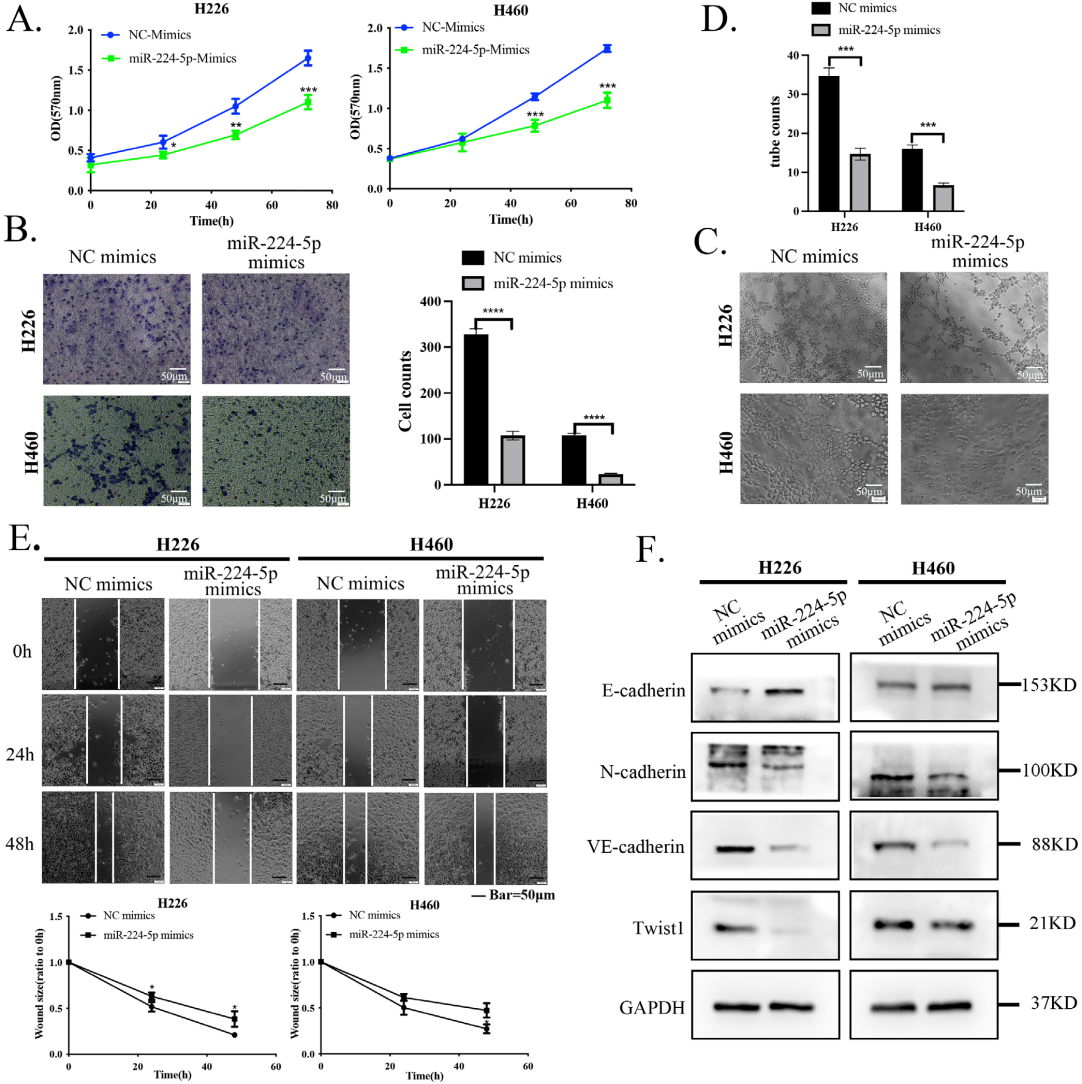
Upregulation of miR‐224‐5p expression inhibits NSCLC progression. (A) Evaluation of the differences in the proliferation ability of H226 and H460 cells after introducing the miR‐224‐5p mimic using the MTT assay. (B) Evaluation of the differences in the invasion of the above four types of cells using the Transwell assay. (C, D) Evaluation of VM formation by H226 and H460 cells and after introducing the miR‐224‐5p mimics in 3D culture assay. (E) Evaluation of the differences in the migration of the four types of cells after treatment with mitomycin C using wound healing assay. (F) The expression levels of E‐cadherin, N‐cadherin, VE‐cadherin, and Twist1 proteins in H226 and H460 cells after introducing the miR‐224‐5p mimics were analyzed by western blotting. The statistical significance was expressed as *p < 0.05; **p < 0.01; and ***p < 0.001. Values are expressed as mean ± standard error.

In contrast, after overexpression of miR‐224‐5p in H226 and H460 cells, we found that the cell proliferation rate, invasion, VM formation, and migration ability were reduced compared to the control group (Figure 3). The above functional results indicate that overexpression of miR‐224‐5p in NSCLC can reduce the growth and spreadability of NSCLC cells. In addition, to further determine that miR‐224‐5p can regulate the progression of NSCLC, we used western blotting to detect the expression of EMT marker proteins after cell treatment. The results indicated that inhibition of miR‐224‐5p in A549 and 95‐D cells significantly increased the expression of Twist1, as well as mesenchymal marker proteins VE‐cadherin and N‐cadherin, while downregulating the expression of the epithelial marker protein E‐cadherin. However, overexpression of miR‐224‐5 in H226 and H460 cells significantly reduced the expression of these proteins (Figures 2F and 3F).

### 3.3 miR‐224‐5p Expression Affects NSCLC Tumor Growth and EMT

**FIGURE 4:**
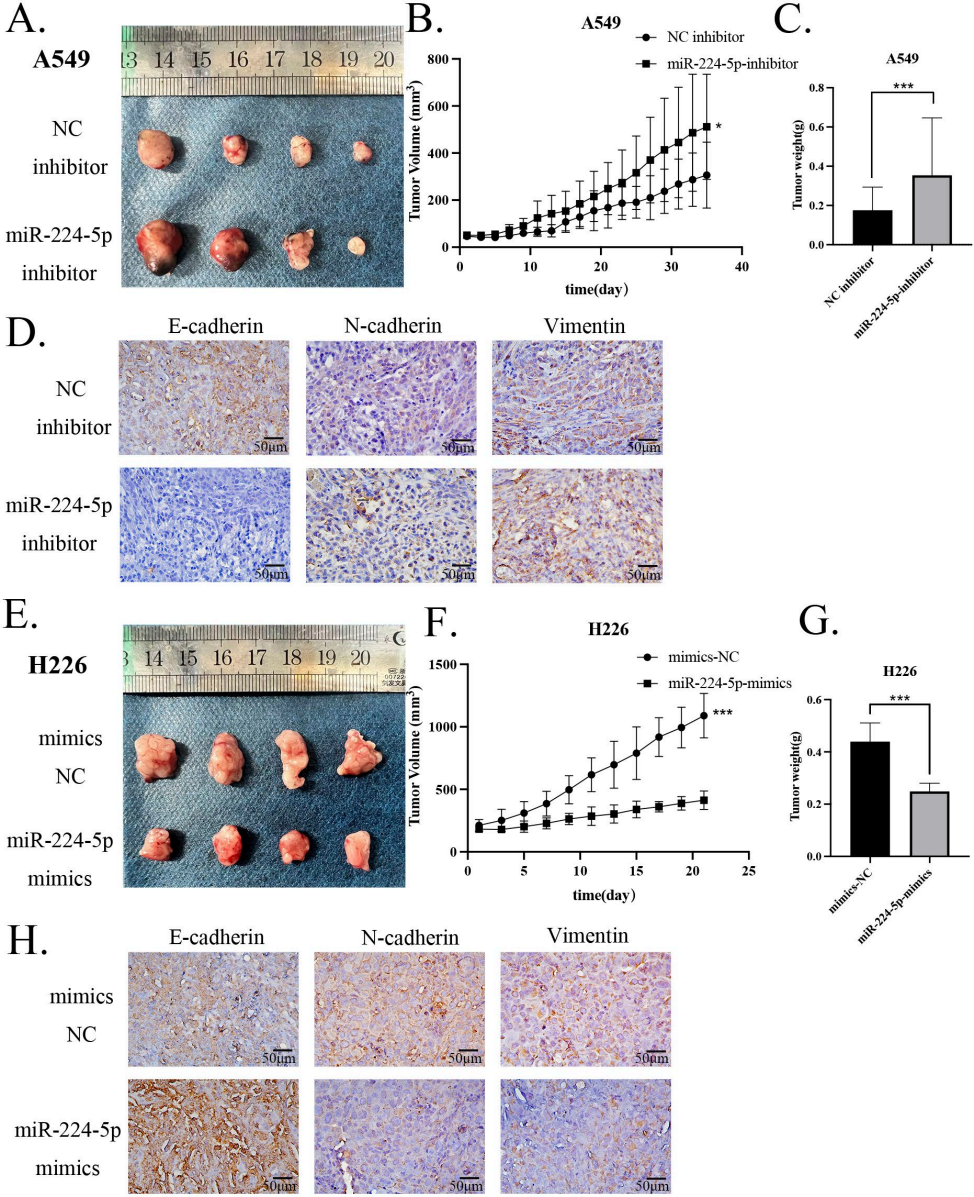
miR‐224‐5p regulates NSCLC tumor growth and EMT in vivo. (A) Representative images of tumor tissues of A549 (control group) and A549 cells stably introduced with miR‐224‐5p inhibitor, 21 days after implantation into nude mice. (B, C) Tumor volume and weight change curves, along with statistical analysis, for A549 cells and those stably introduced with miR‐224‐5p inhibitor. (D) Evaluation of EMT marker expression levels (E‐cadherin, N‐cadherin, and vimentin) in A549 tumor tissues with stable introduction of miR‐224‐5p inhibitor by IHC. (E) Representative images of tumor tissues of H226 cells (control group) and cells stably introduced with miR‐224‐5p mimic, 21 days after implantation into nude mice. (F) Tumor volume change curve and tumor weight statistics for H226 cells and those stably introduced with miR‐224‐5p mimic. (G) Evaluation of EMT marker expression levels (E‐cadherin, N‐cadherin, and vimentin) in H226 tumor tissues with stable introduction of miR‐224‐5p mimic by IHC. The statistical significance was expressed as *p < 0.05; **p < 0.01; and ***p < 0.001. Values are expressed as mean ± standard error.

To further evaluate the effect of miR‐224‐5p on the in vivo growth of NSCLC cells, we established nude mouse xenograft tumor models with the A549 control group. We stably transfected A549 cells with a miR‐224‐5p inhibitor, H226 control cells, and a miR‐224‐5p mimic. By observing and recording tumor volume for 21 days, we observed that the tumor volume of the tumor model established after transfection with the miR‐224‐5p inhibitor was significantly larger than that of the A549 control tumor (Figure 4A,B). Although the tumor weight in the miR‐224‐5p inhibitor group, no significant difference was observed between the two groups (Figure 4C). We also observed that the tumor volume and weight of H226 cells transfected with miR‐224‐5p mimics were significantly reduced compared with the control group (Figure 4E–G). In addition, we further used IHC to evaluate the expression of EMT marker proteins in tumor tissues. The results showed that E‐cadherin expression was downregulated in tumor tissues of A549 cells transfected with the miR‐224‐5p inhibitor, while N‐cadherin and vimentin expressions were upregulated. In contrast, overexpression of miR‐224‐5p in H226 cells promoted the expression of E‐cadherin and inhibited the expression of N‐cadherin and vimentin (Figure 4D,H). These results indicated that miR‐224‐5p affects the growth and EMT of NSCLC tumors in vivo.

### 3.4 miR‐224‐5p Inhibits IL6ST Transcription and Inactivates the JAK2/STAT3 Signaling Pathway

**FIGURE 5:**
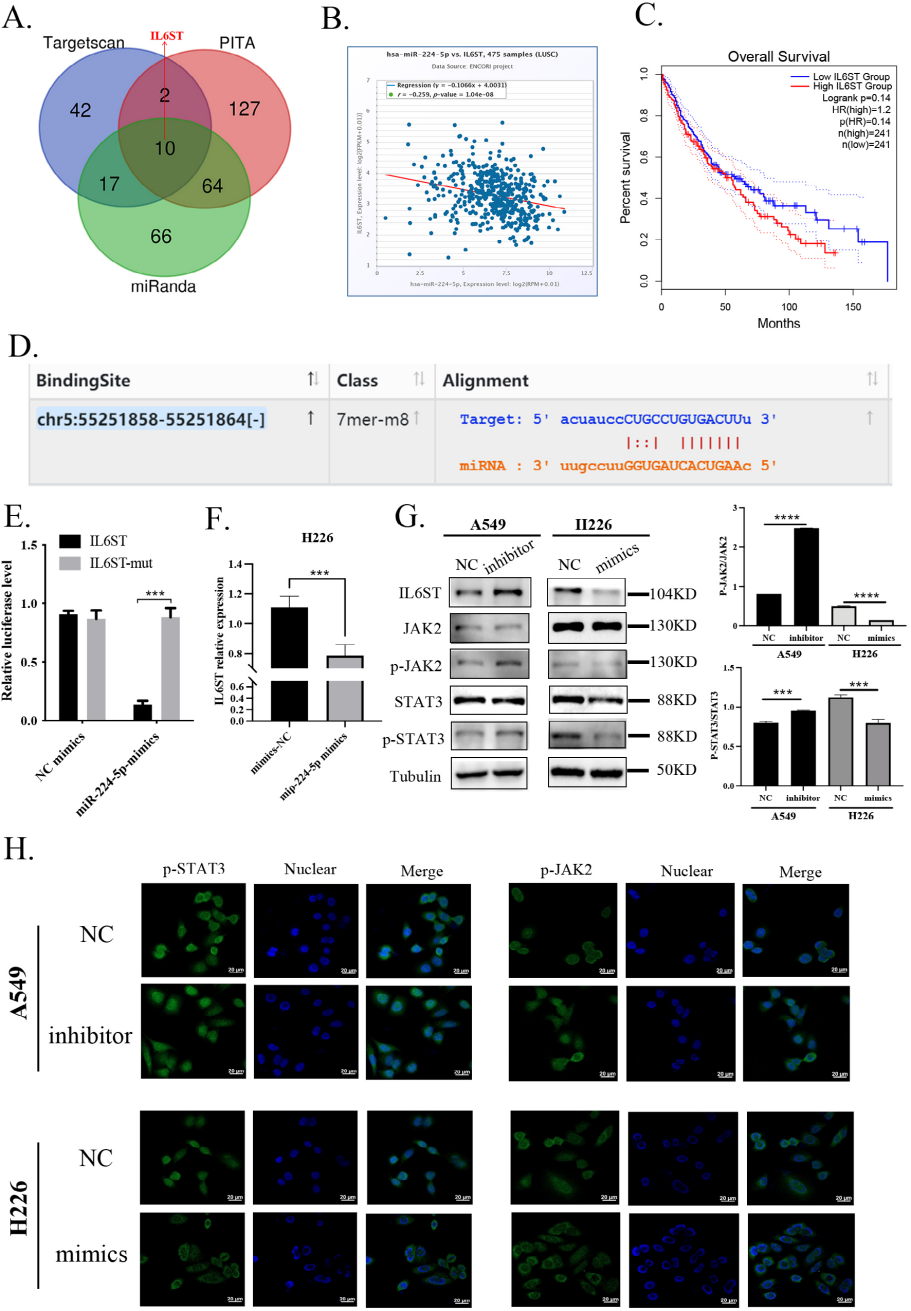
miR‐224‐5p inhibits IL6ST transcription and inactivates the JAK2/STAT3 signaling pathway. (A) Venn diagram showing statistical analysis of miR‐224‐5p–related targets predicted by TargetScan, PITA, and miRanda database. (B) Correlation between IL6ST expression and miR‐224‐5p expression based on database analysis. (C) Survival curve analysis of IL6ST expression in NSCLC using the GEPIA2 database. (D) Prediction of IL6ST binding sites with miR‐224‐5p by database analysis. (E) Luciferase gene reporter assay analysis of the binding of miR‐224‐5p with IL6ST 3′‐UTR fluorescent plasmid and mutant plasmid in H226 cells. (F) RT‐qPCR detection of IL6ST mRNA levels after stable introduction of miR‐224‐5p mimic in H226 cells. (G) Western blot analysis was used to assess the expression levels of IL6ST, JAK2, p‐JAK2, STAT3, and p‐STAT3 proteins in A549 cells, A549 stably introduced with miR‐224‐5p inhibitor, H226 cells, and H226 cells introduced with miR‐224‐5p mimic. (H) Immunofluorescence analysis of the phosphorylated JAK and STAT3 expression and the nuclear localization of p‐STAT3. (H) Immunofluorescence analysis of the phosphorylated JAK and STAT3 expression and the nuclear localization of p‐STAT3. The statistical significance was expressed as *p < 0.05; **p < 0.01; and ***p < 0.001. Values are expressed as mean ± standard error.

Previous studies have not clearly defined the targets and mechanisms of miR‐224‐5p in NSCLC. To identify potential targets, we first used the TargetScan, PITA, and miRanda databases, which collectively predicted 10 common targets: ID3, PDE4DIP, C1orf52, WDR26, IL6ST, RERE, PIK3R3, ARPC5, YOD1, and POGZ (Figure 5A). We further validated these targets using the StarBase database, which confirmed binding sites for miR‐224‐5p in all targets except C1orf52 and RERE, so we excluded them from further analysis (Figure S1A–H, Table S1, and Figure 5D). Next, we analyzed the correlation between miR‐224‐5p and the remaining eight targets in LUSC using the StarBase database. We found a more substantial negative correlation with ID3, PDE4DIP, IL6ST, and PIK3R3 (Figure S1I–P, Figure 5B). Using the cBioPortal and GEPIA2 databases, we further observed that IL6ST had a more pronounced impact on LUSC patient prognosis (Figure S2A–D and Figure 5C). Next, we conducted a literature review on the relationship between ID3, PDE4DIP, IL6ST, PIK3R3, and miR‐224‐5p. We found that PIK3R3 has already been reported as a miR‐224‐5p target in melanoma, where it regulates cancer cell proliferation, migration, and invasion [17]. Additionally, studies have shown miR‐224‐5p is associated with IL6ST expression in hepatocellular carcinoma, suggesting a potential diagnostic role, though the mechanism is not well defined [23]. Therefore, our study focuses on IL6ST to clarify its role as a miR‐224‐5p target in NSCLC.

IL6ST is an activating receptor for IL6 and is associated with the activation of the JAK2/STAT3 signaling pathway. In NSCLC, activation of the JAK2/STAT3 pathway promotes tumor progression. We speculate that miR‐224‐5p may regulate the progression of NSCLC through the IL6/JAK2/STAT3 signaling pathway. To verify our hypothesis, we synthesized the IL6ST luciferase reporter gene plasmids and controlled mutant gene plasmids based on the binding sites to verify the binding between miR‐224‐5p and IL6ST. Through experimental detection, we observed that introducing the miR‐224‐5p mimic into H226 cells could significantly suppress the luciferase activity of IL6ST, while luciferase activity of the mutated IL6ST plasmid remained unaffected (Figure 5E). RT‐qPCR analysis also pointed out that adding miR‐224‐5p mimic could significantly reduce the level of IL6ST mRNA in H226 cells (Figure 5F). Through western blotting analysis, we further found that introducing the miR‐224‐5p inhibitor into A549 cells upregulates the expression levels of IL6ST, as well as p‐STAT3 and p‐JAK2.

In contrast, adding the miR‐224‐5p mimic to H226 cells reduced the expression of IL6ST, as well as phosphorylated STAT3 and JAK2 (Figure 5G). We further pointed out through immunofluorescence experiments that introducing the miR‐224‐5p inhibitor into A549 cells promoted STAT3 and JAK2 phosphorylation, as well as p‐STAT3 nuclear translocation in A549. However, introducing the miR‐224‐5p mimic into H226 cells inhibited STAT3 and JAK2 phosphorylation and prevented p‐STAT3 nuclear translocation (Figure 5H). Our data suggest that miR‐224‐5p may suppress the JAK2/STAT3 signaling pathway activation by targeting IL6ST transcription.

### 3.5 IL6ST Overexpression Reverses the Inhibition of miR‐224‐5p and Activation of the JAK2/STAT3 Pathway in NSCLC

**FIGURE 6:**
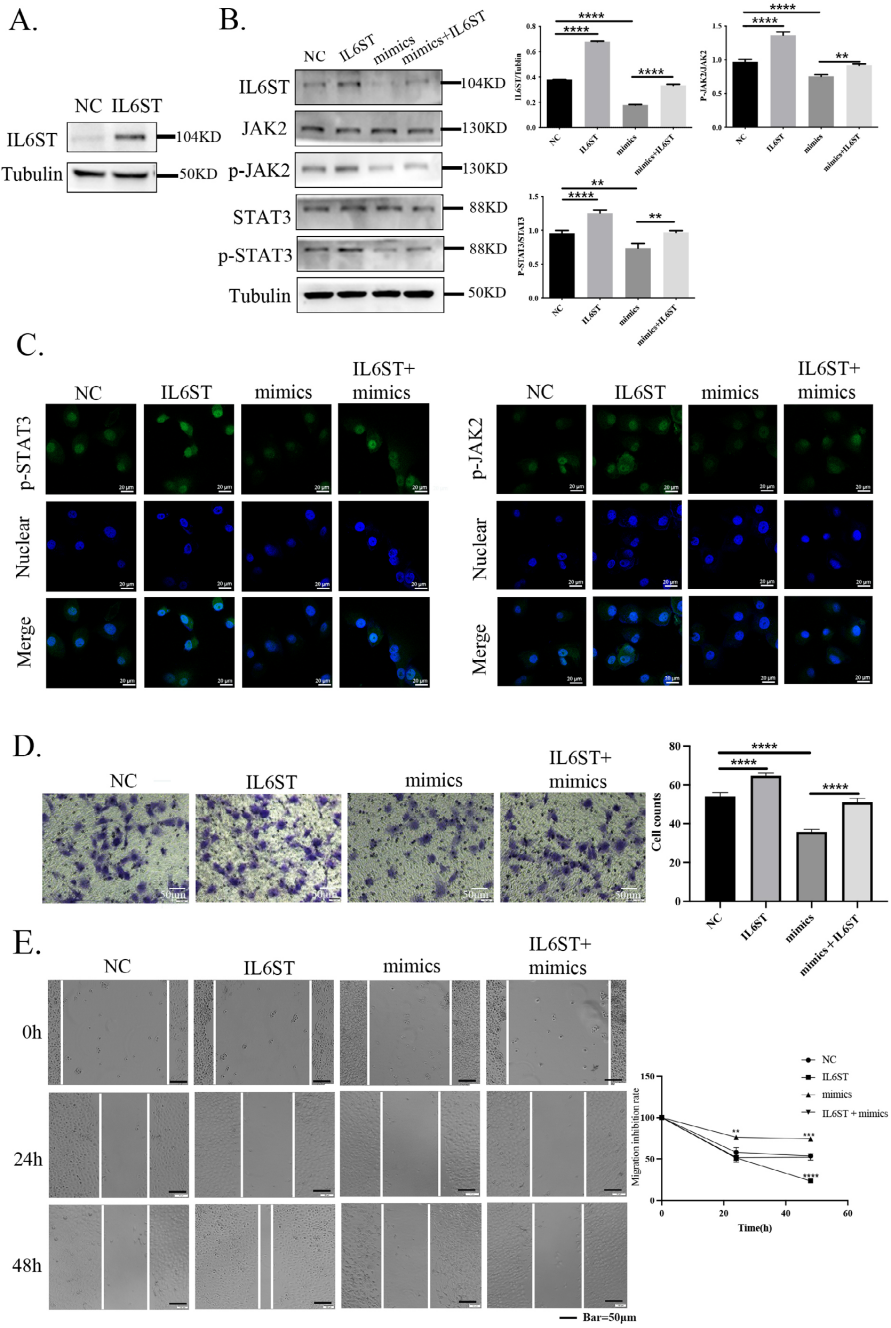
IL6ST overexpression reverses the inhibition of miR‐224‐5p and activation of the JAK2/STAT3 pathway in NSCLC. (A) IL6ST expression levels were detected by western blotting after H226 cells were transfected with IL6ST overexpression plasmid for 48 h. (B) The expression levels of IL6ST, JAK2, STAT3 proteins, and phosphorylated JAK2 and STAT3 proteins were assessed by western blotting in H226 cells under three conditions: overexpression of IL6ST, introduction of miR‐224‐5p mimic, and overexpression of IL6ST followed by introduction of miR‐224‐5p mimic. (C) Detecting the expression levels of phosphorylated JAK2 and STAT3 proteins and the nuclear localization of phosphorylated STAT3 under different cell conditions using an immunofluorescence assay. (D) Evaluation of the difference in the invasion level of H226 cells under different conditions by the Transwell assay. (E) The difference in the migration ability of the four cell types at 0, 24, and 48 h using a wound healing assay. The statistical significance was expressed as *p < 0.05; **p < 0.01; and ***p < 0.001. Values are expressed as mean ± standard error.

To further confirm that miR‐224‐5p suppresses NSCLC by targeting IL6ST, we chose to verify it after overexpressing IL6ST in H226 (Figure 6A). Western blotting and immunofluorescence experiments indicated that overexpression of IL6ST promoted the expression of p‐STAT3 and p‐JAK2 in H226 cells, as well as the translocation of p‐STAT3 to the cell nucleus. In contrast, overexpression of miR‐224‐5p inhibited the expression of p‐STAT3 and p‐JAK2 in H226 cells and prevented the translocation of p‐STAT3 to the cell nucleus. It is worth noting that when we transfected H226 cells with the miR‐224‐5p mimic and then overexpressed IL6ST, the expression of p‐STAT3 and p‐JAK2 was reduced compared with those of overexpressing miR‐224‐5p. Additionally, immunofluorescence results indicated that the cell nuclear translocation of p‐STAT3 was also reduced (Figure 6B,C). We further used Transwell and wound healing assays to detect whether the above treatments affected cell growth. The results indicated that H226 cells showed more robust proliferation and diffusion abilities after overexpressing IL6ST alone. In contrast, the growth migration and diffusion abilities of H226 cells transfected with the miR‐224‐5p mimic were reduced. However, when we simultaneously introduced miR‐224‐5p and overexpressed IL6ST in H226 cells, the proliferation ability of H226 did not show significant differences.

## 4 Discussion

NSCLC is the predominant subtype of lung cancer, responsible for 80%–85% of lung cancer‐related deaths. Traditional treatment approaches primarily involve chemotherapy and radiotherapy following surgical resection [24]. Although advances have been made with the advent of molecular targeted therapies and immunotherapies, the mortality rate from lung cancer continues to rise, highlighting the urgent need for further improvements in diagnosis and treatment.

MicroRNAs act as endogenous inhibitors by interacting with the 3′‐UTR of target gene mRNAs. When microRNAs fully complement the target mRNA 3′‐UTR, they induce cleavage and subsequent degradation. On the other hand, partial binding of microRNAs to the 3′‐UTR can inhibit the translation of target mRNAs [25]. Many microRNAs can alter the expression of target genes by binding to them in cancer cells, thereby either hindering or promoting tumor progression. Studies have shown that in NSCLC, microRNAs are also closely related to processes such as NSCLC cell proliferation [26], apoptosis [27], angiogenesis [28], EMT [29], migration [30], metastasis, and immunosuppression [31]. Therefore, elucidating the role and mechanism of microRNAs in NSCLC will help develop more effective diagnostic and therapeutic strategies.

MiR‐224 is a family of precursor microRNAs widely expressed in humans and a variety of mammals, and is reported to play an important role in regulating tumor progression. However, existing studies have shown that miR‐224‐5p expression levels vary in different types of tumors, thus playing different biological functions. In hepatocellular carcinoma [32], renal cell carcinoma [33], pancreatic mucinous cystadenocarcinoma [34], breast cancer [35], and thyroid papillary carcinoma [36], high expression of miR‐224‐5p promotes tumor progression. In contrast, miR‐224‐5p is downregulated in gastric cancer [37], bone and joint cancer [38], and glioma [19]. Additionally, even within the same tumor type, the expression levels of miR‐224‐5p can vary. For instance, in breast cancer, miR‐224‐5p promotes tumor metastasis by targeting Smad4 [35], but its expression is significantly downregulated in pure mucinous breast carcinoma [39]. Regarding lung cancer, some studies have shown that miR‐224‐5p is upregulated in NSCLC and that exosomal miR‐224‐5p can promote the growth of NSCLC cells [40]. However, other research has shown that miR‐224‐5p is decreased in lung cancer cells and TRG‐AS1 promotes lung cancer cell growth by upregulating the oncogene Smad4 through the sequestration of miR‐224‐5p [41]. Additionally, research suggests that miR‐224‐5p can be sponged by circ‐SHPRH, thereby regulating QKI expression and inhibiting cadmium‐induced bronchial epithelial cell carcinogenesis [42]. However, the specific role of miR‐224‐5p in NSCLC remains unclear and requires further investigation. In our study, we focused on exploring the function and role of miR‐224‐5p in NSCLC. First, we observed that miR‐224‐5p is downregulated in NSCLC patients and cells, and inhibiting miR‐224‐5p promotes the growth of NSCLC cells and tumors, whereas overexpression of miR‐224‐5p showed the opposite effect. We further studied the targets and potential mechanisms of miR‐224‐5p. We investigated its target and found that all three databases predicted IL6ST as a probable target. IL6ST, also known as gp130, is a common signal transduction factor in multiple cytokine receptor complexes, including interleukin 6 (IL6), interleukin 11 (IL11), leukemia inhibitory factor (LIF), and tumor necrosis factor (OSM). When cytokines such as IL6 bind to their specific receptors (such as IL6Ra), IL6ST dimerizes, allowing the intracellular region of IL6ST to bind and activate related Janus kinases (JAKs) [43]. This in turn triggers the activation of downstream signaling pathways, including JAK2/STAT3. Studies have shown that IL6 and IL6ST are overexpressed in NSCLC, and their elevated levels are associated with poor prognosis in patients [44]. The activation of the IL6/IL6ST/JAK/STAT3 pathway contributes significantly to lung cancer development, including tumor growth, angiogenesis, and malignant progression [45]. This study discovered that miR‐224‐5p can bind to IL6ST mRNA, suppress its 3′‐UTR activity and translation, and consequently inhibit the downstream JAK2/STAT3 signaling pathway. This inhibition reduces the invasion and metastasis of NSCLC cells, a finding we also confirmed in animal models. Moreover, overexpression of IL6ST significantly counteracts the suppressive role of miR‐224‐5p on NSCLC progression and the JAK2/STAT3 pathway activation.

In summary, our research demonstrated that miR‐224‐5p is significantly downregulated in NSCLC and identified IL6ST as its direct target. By binding to IL6ST mRNA, miR‐224‐5p reduces IL6ST expression, suppresses the activation of the JAK2/STAT3 signaling pathway, and ultimately inhibits NSCLC cell proliferation and tumor growth. These findings provide valuable insights for the development of novel therapeutic targets and treatment strategies for NSCLC.

## Author Contributions

**Jiao Tian:** investigation, methodology, formal analysis, writing – original draft. **Yiming He and Zihui Zhang:** visualization, formal analysis. **Yuxin Zhu:** formal analysis. **Haixia Ren and Liang Zhang:** validation, data curation. **Lei Li, Wei Li and Weidong Zhang:** validation, visualization. **Ting Xiao:** writing – review and editing, supervision. **Honggang Zhou and Xiaoping Li:** conceptualization, writing – review and editing, supervision, funding acquisition.

## Ethics Statement

All animal care and experimental procedures in this study were performed according to the guidelines approved by the Institutional Animal Care and Use Committee (IACUC) of Nankai University (permit no. SYXK 2014‐0003).

## Conflicts of Interest

The authors declare no conflicts of interest.

## Supporting information


Data S1.

## Data Availability

All experimental and analytical data presented in this study are available in the article or Supporting Information.
