# Supplementary material for: miR‐224‐5p Suppresses Non‐Small Cell Lung Cancer via IL6ST‐Mediated Regulation of the JAK2/STAT3 Pathway
Source: Thorac Cancer. 2025 Jan 22;16(2):e15516. doi: 10.1111/1759-7714.15516 (PMC11751714; doi:10.1111/1759-7714.15516)
Supplement: Supplementary file 1 — Data S1. [file TCA-16-e15516-s001.zip › tca15516-sup-0001-supinfo.docx]

**Supplementary Material**

**Supplementary Figure 1. (A-H): Analysis of the predicted binding sites and binding scores between each target and miR-224-5p, based on StarBase database analysis.**

A. Binding site analysis of target ID3 and miR-224-5p.

B. Binding site analysis of target PDE4DIP and miR-224-5p.

C. Binding site analysis of target WDR26 and miR-224-5p.

D. Binding site analysis of target IL6ST and miR-224-5p.

E. Binding site analysis of target PIK3R3 and miR-224-5p.

F. Binding site analysis of target ARPC5 and miR-224-5p.

G. Binding site analysis of target YOD1 and miR-224-5p.

H. Binding site analysis of target POGZ and miR-224-5p.

**(I-P): Correlation analysis of the predicted targets with miR-224-5p expression in lung squamous cell carcinoma (LUSC) based on StarBase database analysis.**

I. Correlation analysis of target ID3 and miR-224-5p expression.

J. Correlation analysis of target PDE4DIP and miR-224-5p expression.

K. Correlation analysis of target WDR26 and miR-224-5p expression.

L. Correlation analysis of target IL6ST and miR-224-5p expression.

M. Correlation analysis of target PIK3R3 and miR-224-5p expression.

N. Correlation analysis of target ARPC5 and miR-224-5p expression.

O. Correlation analysis of target YOD1 and miR-224-5p expression.

P. Correlation analysis of target POGZ and miR-224-5p expression.

**Table1**

|  | **Targets** | **Target-directed miR-224-5p**  **Degradation (TDMD) Score** |
| --- | --- | --- |
| 1 | ID3 | 0.4298 |
| 2 | PDE4DIP | 1.3391 |
| 3 | C1orf52 | No Results |
| 4 | WDR26 | 1.0894 |
| 5 | IL6ST | 0.727 |
| 6 | RERE | No Results |
| 7 | PIK3R3 | 0.5934 |
| 8 | ARPC5 | 0.842 |
| 9 | YOD1 | 0.4216 |
| 10 | POGZ | 0.4019 |

**Table 1. Target Genes of miR-224-5p and Statistics of Target-Directed miR-224-5p Degradation (TDMD) Score.**

**Supplementary Figure 2. (A-D): Analysis of the impact of predicted targets on survival in LUSC patients based on the Cbioportal database.**

1. Survival analysis of target ID3 in LUSC patients.

B. Survival analysis of target PDE4DIP in LUSC patients.

C. Survival analysis of target IL6ST in LUSC patients.

D. Survival analysis of target PIK3R3 in LUSC patients.
