# Supplementary figures and images for: miR‐224‐5p Suppresses Non‐Small Cell Lung Cancer via IL6ST‐Mediated Regulation of the JAK2/STAT3 Pathway
Source: Thorac Cancer. 2025 Jan 22;16(2):e15516. doi: 10.1111/1759-7714.15516 (PMC11751714; doi:10.1111/1759-7714.15516)

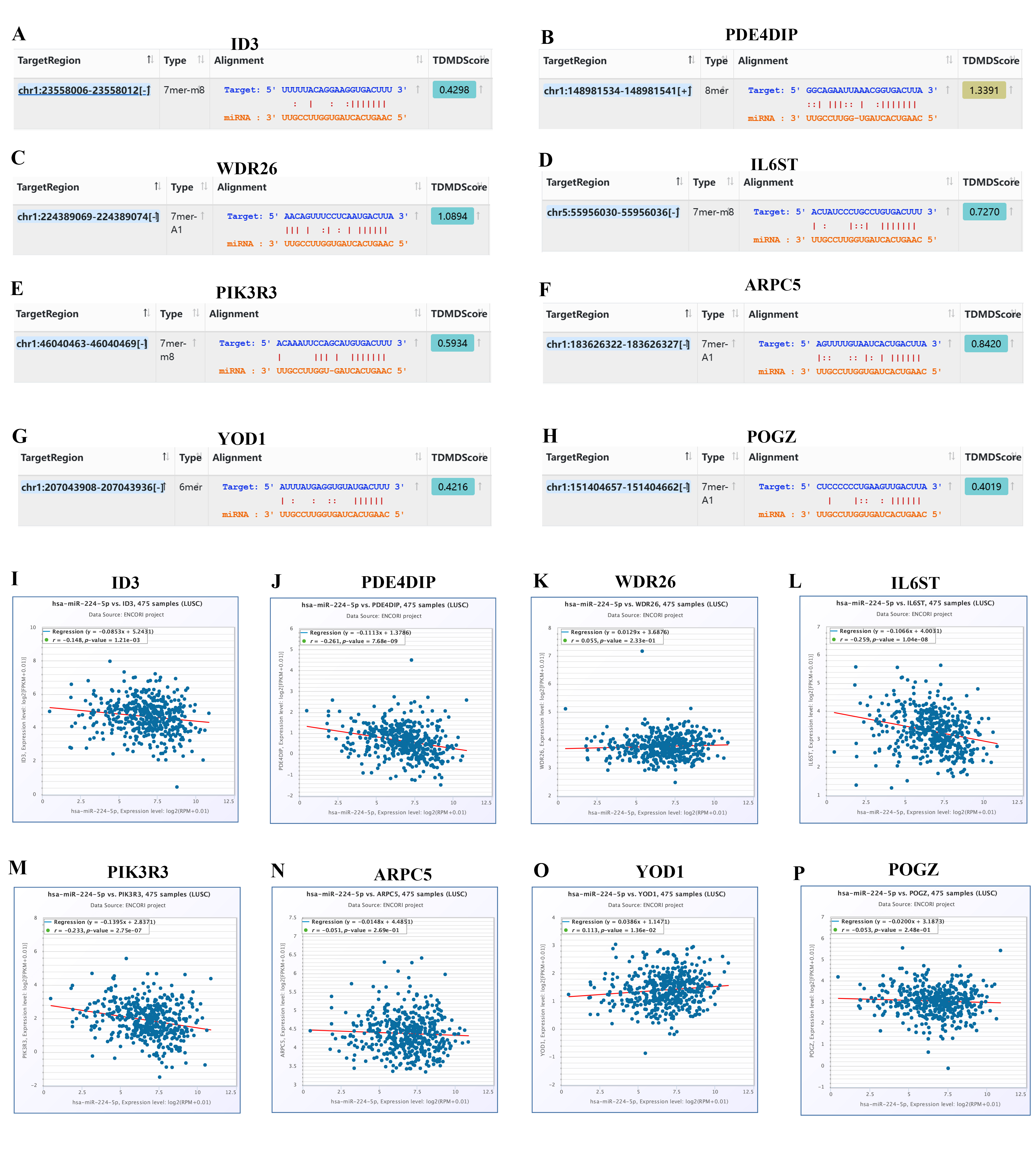

Supplement: Supplementary file 1 — Data S1. [file TCA-16-e15516-s001.zip › Supplementary Figure 1.jpg]

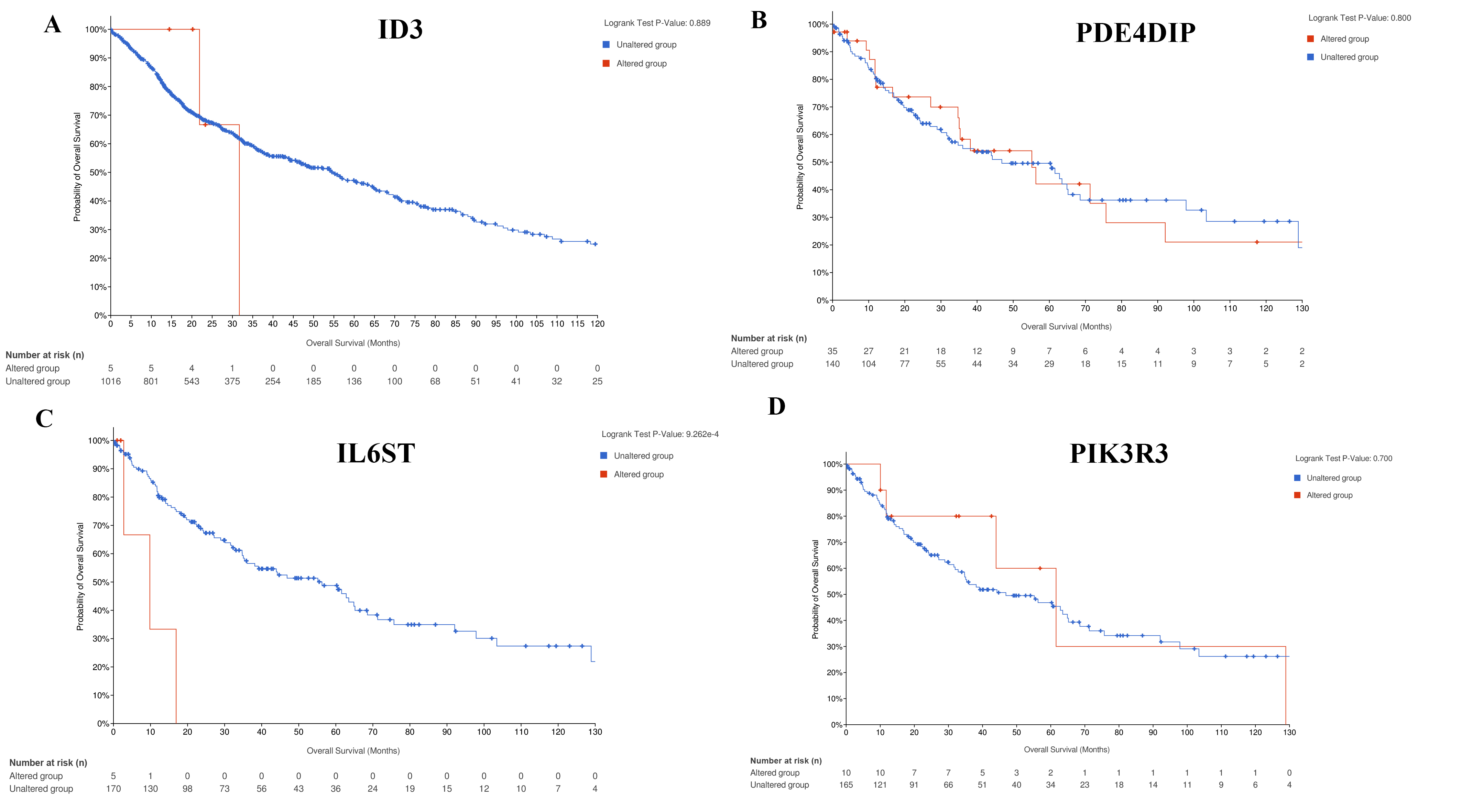

Supplement: Supplementary file 1 — Data S1. [file TCA-16-e15516-s001.zip › Supplementary Figure 2.jpg]
